# KMT2C Knockdown impairs neuronal differentiation in human induced pluripotent stem cell-derived neural stem cells

**DOI:** 10.17912/micropub.biology.002149

**Published:** 2026-05-11

**Authors:** Masaya Ogawa, Shotaro Kawano, Sayaka Katayama, Naoto Ikeda, Rei Endo, Tomoki Mita, Yuuri Ikeuchi, Hotaka Fukushima, Daiki Miura, Takanobu Nakazawa

**Affiliations:** 1 Tokyo University of Agriculture, Tokyo, 156-8502, Japan

## Abstract

KMT2C mutations are strongly associated with autism spectrum disorder (ASD). Whereas multiplexed CRISPR screens show complete KMT2C knockout impairs neuronal differentiation, mixed-genotype pools cannot exclude non-autonomous paracrine effects. Furthermore, the functional effect of partial loss of function remains unclear. Here, we targeted KMT2C via shRNA knockdown in single-genotype cultures of human induced pluripotent stem cell-derived neural stem cells. Reduced KMT2C expression decreased the neuronal markers MAP2, HuC/D, and DCX. Our results demonstrate that partial, strictly cell-autonomous KMT2C insufficiency impairs neuronal stem cell differentiation into neurons, modeling the haploinsufficiency observed in patients to reveal a neurodevelopmental pathogenesis for ASD.

**Figure 1. KMT2C is required for differentiation of human NSCs into neurons:** (A) Schematic illustration of the experimental workflow, including shRNA-mediated KMT2C knockdown in human iPSC-derived neural stem cells and subsequent neuronal differentiation. (B) Representative immunofluorescence images showing HuC/D (magenta) and GFP (green) in differentiated cells at Day 2 of differentiation. Nuclei were counterstained with Hoechst 33258 (blue). Yellow arrowheads indicate double-positive cells. Scale bars, 20 µm. (C) Quantification of the percentage of HuC/D-positive cells among the GFP-positive (transfected) cells at Day 2. A significant reduction in HuC/D-positive neurons was observed in KMT2C-knockdown cells compared to the control. Data are presented as mean ± SEM from five independent experiments (shControl: n = 5; shKMT2C-1: n = 5; shKMT2C-2: n = 5). In this representative analysis, a total of 3,300 (shControl), 2,271 (shKMT2C-1), and 3,074 (shKMT2C-2) GFP-positive cells were evaluated. (D) Relative mRNA expression levels of neuronal differentiation markers ( *ELAVL3* , *ELAVL4* , *MAP2* , and *DCX* ) determined by qPCR at Day 2. Expression levels were normalized to *ACTB* . All markers showed significant downregulation upon KMT2C knockdown. Data are presented as mean ± SEM (n = 3 per group). Statistical significance was analyzed using a one-way ANOVA followed by Tukey's *post hoc* test (** *P* < 0.01, *** *P* < 0.001). Illustrations were created using BioRender.com.

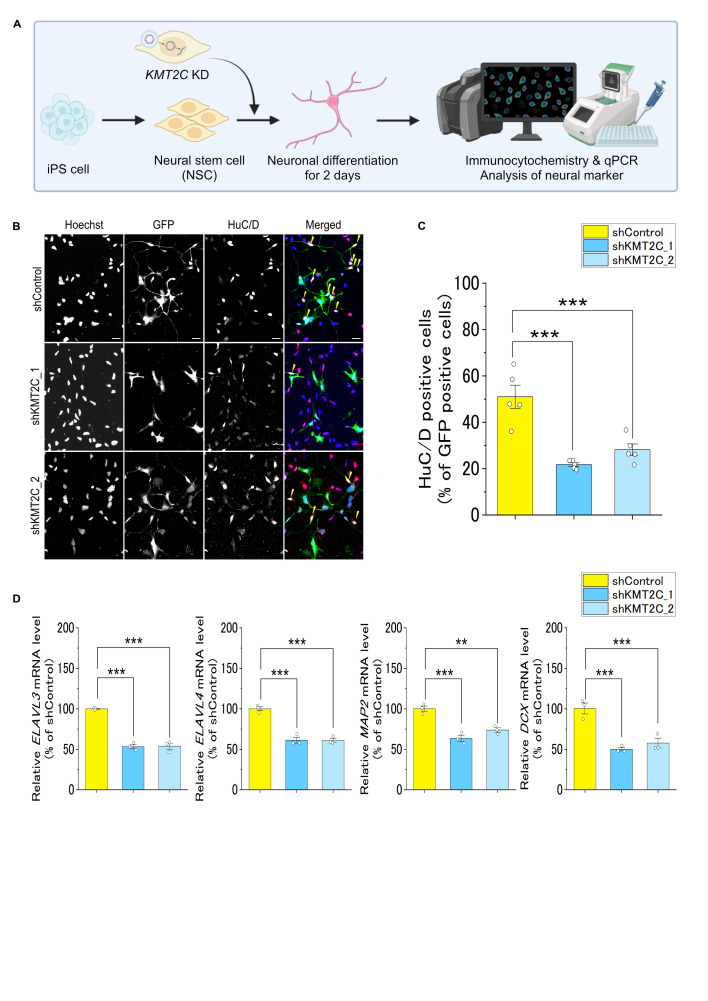



## Description

Autism spectrum disorder (ASD) is a complex neurodevelopmental condition characterized by a highly heterogeneous genetic architecture (Lord et al., 2020). Recent large-scale whole-exome sequencing studies have implicated rare disruptive mutations in chromatin regulators as the genetic drivers of ASD, with *KMT2C* (also known as *MLL3* ) emerging as a high-risk gene (Satterstrom et al., 2020; Fu et al., 2022). *KMT2C* encodes a core histone H3K4 mono-methyltransferase within the conserved COMPASS (complex proteins associated with Set1) family, and it contributes to the establishment of enhancer landscapes during embryonic development and lineage specification (Hu et al., 2013; Rao and Dou, 2015). Meanwhile, induced pluripotent stem cell (iPSC) technology has become an indispensable tool for investigating the molecular and cellular pathogenesis underlying ASD. iPSC-based approaches provide a human-specific platform for evaluating how targeted genetic modifications introduced into healthy control lines impair neurodevelopmental processes, thereby bridging the gap between mouse models and human clinical research (Nakazawa, 2022).

To evaluate the functional effect of KMT2C loss, a recent study, using a multiplex platform based on human embryonic stem cells, demonstrates that the complete knockout of KMT2C severely impairs neuronal differentiation (Cederquist et al., 2020). Although this multiplex platform provided crucial mechanistic insights, its pooled design raised two important questions. First, because diverse mutant cells are co-cultured in mixed-genotype pools, it is difficult to exclude paracrine effects. Accordingly, the impaired neuronal differentiation could have arisen from either an intrinsic genetic defect (cell-autonomous) or altered extracellular signals originating from neighboring mutant cells (non-autonomous). Second, because ASD-associated *KMT2C* mutations typically result in a heterozygous loss of function (haploinsufficiency), whether a partial reduction in KMT2C levels is sufficient to impair neuronal differentiation remains unclear. To address these issues, we employed a targeted knockdown (KD) approach to establish single-genotype cultures of KMT2C-deficient human iPSC-derived neural stem cells (NSCs), using two independent short hairpin RNAs (shRNAs) to evaluate the cell-autonomous effect of KMT2C insufficiency.

First, we validated the knockdown efficiency in human iPSC-derived NSCs. An quantitative RT-PCR (qPCR) analysis confirmed a significant reduction in *KMT2C* mRNA expression levels in both KMT2C-deficient lines relative to those in controls (shKMT2C_1, 71.4 ± 3.10 (% of controls); shKMT2C_2, 78.2 ± 1.72 (% of controls). Following validation, the cells were subjected to neuronal differentiation for 2 days ( Figure 1A ). To assess neuronal differentiation, we performed immunofluorescence staining for the RNA-binding proteins HuC/D, a well-established marker of neuronal differentiation that defines an early post-mitotic neuronal identity (Okano and Darnell, 1997). Quantitative analysis confirmed a significant reduction in the percentages of HuC/D-positive cells in both KMT2C-KD lines compared to that in the control ( Figure 1B, C). Furthermore, qPCR revealed a significant decrease in the mRNA expression of *ELAVL3/4* (encoding HuC/D) and microtubule-associated protein 2 ( *MAP2* ), a standard marker of dendritic outgrowth and structural maturation (Dehmelt and Halpain, 2005) ( Figure 1D ). Consistent with the results of previous multiplex screens (Cederquist et al., 2020), doublecortin ( *DCX* ) mRNA expression, an established marker of neurogenesis and early neuronal differentiation, was also significantly reduced in the KMT2C-KD lines ( Figure 1D ).

Several limitations of this study should be noted. First, whereas MAP2 and HuC/D are reliable markers of neuronal identity, we did not determine whether KD-affected cells eventually achieved functional synaptic integration. Future electrophysiological studies are required to determine the physiological consequences. Second, given the role of KMT2C in enhancer–promoter communication (Hu et al., 2013; Kubo et al., 2024), further transcriptomic profiling is essential to identify the specific gene networks disrupted by *KMT2C* insufficiency. Finally, we did not perform a genetic rescue experiment to definitively confirm on-target specificity. Because KMT2C is an exceptionally large protein (~540 kDa), the construction and delivery of a full-length exogenous expression vector is technically unfeasible in the hPSC model. However, our use of two independent shRNA sequences strongly mitigates the likelihood of off-target effects.

In conclusion, our results demonstrate that a partial reduction in KMT2C expression, mimicking the functional deficit observed with haploinsufficiency, is sufficient to impair neuronal differentiation. The observation of these phenotypes in separate single-genotype cultures suggests a cell-autonomous defect. Altogether, our data support the indispensable role for KMT2C in the neuronal differentiation from NSCs into neurons, providing a cellular basis for the neurodevelopmental pathogenesis of ASD.

## Methods

***Cell line***

We used the human iPSC line 201B7, which was provided by the RIKEN Bioresource Center (Tsukuba, Japan).

***Culture of iPSC line***

iPSCs were cultured in StemFit medium (AK02N, Ajinomoto, Tokyo, Japan) in 6-well plates (353046, Corning, NY, USA). The wells were pre-coated with either Matrigel hESC-Qualified Matrix (354277, Corning) diluted 1:50 in DMEM containing High Glucose and L-Glutamine (043-30085, Wako, Osaka, Japan) at 1 mL/well or iMatrix-511 silk (892021, Nippi, Tokyo, Japan) at 0.5 µg/cm². Cells were plated at a density of 1–6 × 10⁴ cells/well and routinely passaged every 5–8 days. To facilitate cell survival during passaging, the ROCK inhibitor Y-27632 (10 µM; 034-24024, Wako) was added to the culture medium overnight. Cell dissociation was carried out using a 1:1 mixture of TrypLE Select enzyme (12563-011, Thermo Fisher Scientific, CA, USA) and 0.5 mM EDTA in PBS (15575-038, Thermo Fisher Scientific).

***Neuronal induction***

To generate NSCs, we applied a dual SMAD inhibition protocol using the PSC Neural Induction Medium (A1647801, Thermo Fisher Scientific). Briefly, iPSCs were seeded in Matrigel-coated 6-well plates at a density of 1 × 10⁶ cells/well. After 24 h, the medium was changed to PSC Neural Induction Medium (2 mL/well) and the cultures were maintained for approximately 1 week. The cells were then harvested using Accutase (AT104, Innovative Cell Technologies, CA, USA) and transferred to new plates at 1 × 10⁶ cells/well in Neural Expansion Medium. This expansion medium was formulated using Neurobasal Medium (21103-049, Thermo Fisher Scientific), Advanced DMEM/F-12 (12634-028, Thermo Fisher Scientific), and a Neural Induction Supplement at a volumetric ratio of 49:49:2. To promote cell viability after replating, 10 µM Y-27632 was added for 24 h. The resulting NSCs were collected after approximately 1 week of expansion for downstream analyses.

***Quantitative RT-PCR (qPCR)***

Total RNA was extracted from the cultured cells using the PureLink RNA Mini Kit (12183025, Thermo Fisher Scientific). The isolated RNA was then reverse-transcribed into cDNA using SuperScript III Reverse Transcriptase (18080085, Thermo Fisher Scientific). For qPCR, we used the TB Green Premix Ex Taq II (Tli Rnase H Plus) master mix (RR820D, Takara Bio, Shiga, Japan) and analyzed the reactions using a CFX96 real-time PCR detection system (Bio-Rad Laboratories, CA, USA). Relative mRNA expression levels were calculated employing the ΔΔ ^Ct^ method. Data from technical triplicates were normalized to the expression of the endogenous control, *ACTB* . The primer sequences eused in these assays are listed in the Reagents and Tools Table.

***shRNA-mediated knockdown of KMT2C***

*KMT2C* gene silencing in iPSC-derived NSCs was achieved through transfection with MISSION shRNA plasmid vectors (Sigma-Aldrich, MO, USA). We employed two distinct shRNA sequences targeting *KMT2C* , specifically shKMT2C-1 (TRCN0000008742; 5′-GAGGCGATTCAACACACCATT-3′) and shKMT2C-2 (TRCN0000008745; 5′-CCAGATACTTTAGTTGATGAA-3′), along with a non-targeting MISSION shRNA as a negative control construct. NSCs were plated in 6-well plates at a density of 1.5 × 10⁶ cells/well and incubated overnight. The plasmids were introduced into the cells using the FuGENE HD Transfection Reagent (E2311, Promega, WI, USA) following the manufacturer’s instructions. To minimize potential cytotoxicity, the transfection mixture was completely replaced with fresh culture medium at 5 h post-transfection. Following a 48 h incubation, transfected cells were selected by adding puromycin (2 µg/mL; A11138-03, Gibco) to the medium for 24 h. The efficiency of *KMT2C* depletion was confirmed via qPCR prior to differentiation assays.

***Neuronal differentiation from NSCs into neurons***

To induce neuronal differentiation, NSCs were harvested with Accutase and seeded into 12-well plates (353043, Corning) at a density of 6 × 10⁵ cells/well. Prior to cell plating, the wells were sequentially coated with poly-L-ornithine (P3655, Sigma-Aldrich, MO, USA) and a PBS mixture containing 6.67 µg/mL of human fibronectin (33016-015, Thermo Fisher Scientific) and 6.67 µg/mL of mouse laminin (23017-015, Thermo Fisher Scientific) at 1 mL/well. The cells were maintained in a neural differentiation medium based on the BrainPhys Neuronal Medium (ST-05790; STEMCELL Technologies, BC, Canada). This medium was enriched with 2% B-27 supplement (17504-044, Thermo Fisher Scientific), 1% N2 supplement (141-09041, Wako), 200 µM L-ascorbic acid (A4403, Sigma-Aldrich), 1 mM bucladesine sodium (029-16383, Wako), 20 ng/mL recombinant human BDNF (248-BD/CF, R&D Systems, MN, USA), 20 ng/mL recombinant human GDNF (212-GD/CF, R&D Systems), 500 ng/mL mouse laminin, and 1 µM DAPT (043-33581, Wako).

***Immunocytochemistry***

For immunocytochemical analyses, neurons were cultured on 12-mm cover glasses (No.1-S, Matsunami Glass, Osaka, Japan) coated with poly-L-ornithine, fibronectin, and laminin, as described previously herein. The cells were rinsed with PBS and fixed in 4% paraformaldehyde. Permeabilization was performed using 0.1% Triton X-100 (T9284, Sigma-Aldrich), followed by a blocking step with 4% goat serum (16210-064, Thermo Fisher Scientific). The samples were then incubated overnight at 4°C with the following primary antibodies: mouse anti-HuC/HuD (1:100; A-21271, Thermo Fisher Scientific) and chicken anti-GFP (1:500; ab13970, Abcam). The next day, the cells were washed and incubated with Alexa Fluor 555-conjugated goat anti-mouse IgG (1:250; A-21424, Invitrogen) and Alexa Fluor 488-conjugated goat anti-chicken IgY (1:250; A11039, Invitrogen) secondary antibodies for 2 h at room temperature. Nuclear counterstaining was performed using Hoechst 33258 dye (1:250; 382061-S, Merck Millipore, CA, USA). Coverslips were mounted onto the slides using Fluoromount (K024; Diagnostic BioSystems, CA, USA). Image acquisition and subsequent analyses were performed using a BZ-X810 fluorescence microscope equipped with BZ-X800 Analyzer software (KEYENCE, Tokyo, Japan). For the quantification of neuronal differentiation efficiency, images were randomly acquired from 10 independent fields of view per well (totaling 50 fields of view across 5 wells per group). The numbers of GFP-positive cells and HuC/D double-positive cells were counted automatically using the BZ-X800 Analyzer software. The positivity criteria were strictly defined by the spatial overlap of Hoechst 33258 (blue), GFP (green), and HuC/D (magenta) signals, which visually co-localized to appear white. In the analyzed representative experiment, the total numbers of evaluated GFP-positive cells were 3,300 for shControl, 2,271 for shKMT2C_1, and 3,074 for shKMT2C_2.

***Statistical analysis***

To evaluate the statistical significance of phenotypic and gene expression differences between control and KMT2C-KD samples, we performed a one-way ANOVA followed by Tukey’s *post hoc* test. Asterisks indicate statistical significance. **P < 0.01, and ***P < 0.001.

## Reagents

**Table:** 

**Reagent/Tool**	**Provider**	**Catalog/Identifier**
**Cell Line**		
201B7 (Human iPSC line)	RIKEN BRC	Cat# HPS0063; RRID: CVCL_A324
**shRNA Vectors**		
MISSION® shRNA targeting human KMT2C (shKMT2C-1)	Sigma-Aldrich	TRC clone ID: TRCN0000008742
MISSION® shRNA targeting human KMT2C (shKMT2C-2)	Sigma-Aldrich	TRC clone ID: TRCN0000008745
MISSION® non-target shRNA control vector	Sigma-Aldrich	Cat# SHC002
**Antibodies**		
Mouse anti-HuC/HuD monoclonal antibody	Thermo Fisher Scientific	Cat# A-21271; RRID: AB_221448
Chicken anti-GFP polyclonal antibody	Abcam	Cat# ab13970; RRID: AB_300798
Alexa Fluor 488-goat anti-chicken IgY	Invitrogen	Cat# A11039; RRID: AB_2534096
Alexa Fluor 555-goat anti-mouse IgG	Invitrogen	Cat# A-21424; RRID: AB_141780
**qPCR Primers (Human)**		
ACTB forward primer	FASMAC	Sequence: 5'-CCAACCGCGAGAAGATGA-3'
ACTB reverse primer	FASMAC	Sequence: 5'-CCAGAGGCGTACAGGGATAG-3'
MAP2 forward primer	FASMAC	Sequence: 5'-CCTGTGTTAAGCGGAAAACC-3'
MAP2 reverse primer	FASMAC	Sequence: 5'-AGAGACTTTGTCCTTTGCCTGT-3'
ELAVL3 (HuC) forward primer	FASMAC	Sequence: 5'-CACCTCTACCAGTCATCCGC-3'
ELAVL3 (HuC) reverse primer	FASMAC	Sequence: 5'-ATGAGCGACAGGGGACTCTT-3'
ELAVL4 (HuD) forward primer	FASMAC	Sequence: 5'-GACCCAGAAGGAACTGGAGC-3'
ELAVL4 (HuD) reverse primer	FASMAC	Sequence: 5'-CCCTCTGGACACTCCTGTGA-3'
DCX forward primer	FASMAC	Sequence: 5'-AGCCATCAAACTGGAGACCG-3'
DCX reverse primer	FASMAC	Sequence: 5'-TCAGCTGGAGACTTGCTTCG-3'
